# Pharmacokinetics of metronomic temozolomide in cerebrospinal fluid of children with malignant central nervous system tumors

**DOI:** 10.1007/s00280-022-04424-4

**Published:** 2022-03-30

**Authors:** Sören Büsker, Walter Jäger, Stefan Poschner, Lisa Mayr, Valentin Al Jalali, Johannes Gojo, Amedeo A. Azizi, Sami Ullah, Muhammad Bilal, Lobna El Tabei, Uwe Fuhr, Andreas Peyrl

**Affiliations:** 1 Center for Pharmacology, Faculty of Medicine, University Hospital Cologne, University of Cologne, Cologne, Germany; 2 Department of Pharmaceutical Sciences, Division of Pharmaceutical Chemistry, University of Vienna, Vienna, Austria; 3 Department of Pediatric and Adolescent Medicine, Medical University of Vienna, Comprehensive Center for Pediatrics, Medical University of Vienna, Waehringer Guertel 18-20, 1090 Vienna, Austria; 4 Department of Clinical Pharmacology, Medical University of Vienna, Vienna, Austria; 5 Department of Clinical Pharmacy, Institute of Pharmacy, University of Bonn, Bonn, Germany

**Keywords:** Temozolomide, Pediatrics, Pharmacokinetic, Central nervous system, Cerebrospinal fluid

## Abstract

**Purpose:**

Although temozolomide is widely used in the treatment of childhood central nervous system (CNS) tumors, information on its pharmacokinetic profile in the brain or cerebrospinal fluid (CSF) is sparse. This study aimed at investigating whether measurable and clinically relevant concentrations of temozolomide are reached and maintained in CSF for continuous oral administration in pediatric patients. A population pharmacokinetic model was developed to quantify CSF penetration of temozolomide.

**Methods:**

Eleven pediatric CNS tumor patients (aged 4–14 years) treated with oral temozolomide using a metronomic schedule (24–77 mg/m^2^/day) were included. Temozolomide concentrations in 28 plasma samples and 64 CSF samples were analyzed by high-performance liquid chromatography. Population pharmacokinetic modeling and simulations were performed using non-linear mixed effects modeling (NONMEM 7.4.2).

**Results:**

Median temozolomide concentrations in plasma and CSF were 0.96 (range 0.24–5.99) µg/ml and 0.37 (0.06–1.76) µg/ml, respectively. A two-compartment model (central/plasma [1], CSF [2]) with first-order absorption, first-order elimination, and a transit compartment between CSF and plasma adequately described the data. Population mean estimates for clearance (CL) and the volume of distribution in the central compartment (V_c_) were 3.29 L/h (95% confidence interval (CI) 2.58–3.95) and 10.5 L (8.17–14.32), respectively. Based on simulations, we found a median area under the concentration vs. time curve ratio (AUC_CSF_ / AUC_plasma_ ratio) of 37%.

**Conclusion:**

Metronomic oral temozolomide penetrates into the CSF in pediatric patients, with even higher concentration levels compared to adults.

**Supplementary Information:**

The online version contains supplementary material available at 10.1007/s00280-022-04424-4.

## Introduction

Tumors of the central nervous system (CNS) constitute the largest group of solid neoplasms in children, developing in approximately 5.6 children per 100.000 per year [1].

Standard treatment of malignant childhood CNS tumors consists of maximal safe resection, followed by irradiation and / or chemotherapy. Treatment of these tumors is complicated by the tendency to leptomeningeal dissemination. Chemotherapeutic drug concentrations within the CNS depend on multiple factors, including the permeability of the blood–brain-barrier (BBB) to the chemotherapeutic agent [2].

Temozolomide, an imidazotetrazinone methylating agent, has demonstrated ubiquitous distribution into all tissues, including the CNS [3–5], and is widely used in treatment of malignant glioma in children, frequently in combination with other drugs [6].

Temozolomide is rapidly absorbed after oral administration with almost 100% bioavailability, and its plasma pharmacokinetics is linear and predictable, both in adults and children [7–10].

Temozolomide is a pro-drug for methyl triazene (MITC), which is the final active methylating compound [3].

Although temozolomide is widely used in treatment of CNS tumors, there is only scarce information on the concentration profile of temozolomide in the brain or cerebrospinal fluid (CSF) after oral administration [5, 11, 12]. It has been shown that the area under the concentration vs. time curve in CSF (AUC_CSF_) corresponded to approximately 20% of the area under the concentration vs. time curve in plasma (AUC_plasma_) in adult patients [5].

The recommended dosing schedule of temozolomide is concomitant to local irradiation with 75 mg/m^2^/day, followed by cycles of adjuvant temozolomide with 150–200 mg/m^2^/day for 5 days [13, 14], leading to a median survival in glioblastoma of 14.6 months in adults [13].

The rationale behind the maximum tolerated dose is to use the highest tolerated concentration of chemotherapy to directly kill tumor cells, but in the inevitable treatment-free period resistance might emerge. In the recurrent setting, continuous application of chemotherapy in lower doses without a treatment-free period is a frequently used treatment approach, where vascular formation is inhibited, thereby preventing tumor progression indirectly [15, 16].

Additionally, repeated treatment with low dose temozolomide is able to cause cytotoxicity through apoptosis, cytostasis through cellular senescence, and DNA double-strand breaks in glioblastoma cells [17].

Responses to temozolomide provided in low continuous dosing have been documented in second-line treatment, leading to median overall survival of nine months in recurrent glioblastoma and 28 months in recurrent neuroendocrine neoplasms [18, 19]. In case of temozolomide, such a metronomic schedule consists of a significantly lower daily dose, typically at 25–90 mg/m^2^/day on a continuous basis, thereby exhibiting an antiangiogenic effect [20, 21].

Although temozolomide is readily applied in the treatment of different childhood brain tumors, there are so far no data on the pharmacokinetic profile of temozolomide in CSF in children after oral administration.

The aim of our study was to establish a pharmacokinetic profile of temozolomide after oral administration in the pediatric population. A population pharmacokinetic model was developed to describe the concentration–time profile of temozolomide in plasma and CSF, and to quantify CSF penetration of temozolomide in our patient population.

## Patients and methods

### Patients and treatment

Pediatric patients with a histologically proven diagnosis of a malignant brain tumor and leptomeningeal dissemination or the risk of such dissemination, who received temozolomide and intraventricular therapy were included in this prospective study. All patients had a subcutaneous indwelling intraventricular catheter (Ommaya reservoir) in place. Other eligibility criteria were (1) life expectancy of at least 8 weeks; (2) written informed consent from the patients and/or parents; (3) serum creatinine < 1.5 mg/dL; (4) total serum bilirubin less than 2.0 mg/dL and alanine aminotransferase less than five times the upper limit of normal; and (5) platelet count above 40,000/mm^3^ within 48 h before the first treatment.

The exclusion criteria were (1) contraindication for administration of temozolomide (e.g., severe uncontrolled infection or inadequate hematological or hepatorenal function); (2) contraindication for administration of intraventricular chemotherapy (e.g., evidence of obstructive hydrocephalus or compartmentalization of CSF flow); (3) pregnancy or breast feeding.

The study protocol was approved by the ethics committee of the Medical University of Vienna and registered in the European Clinical Trials Database (EudraCT number 2015-002,675-19). Patients received temozolomide daily orally in a continuous metronomic schedule. The medical history, a physical examination, and laboratory studies were obtained before treatment. Baseline head and spinal magnetic resonance imaging (MRI), with and without contrast enhancement, was obtained before intraventricular treatment. Cell counts, microbiology, cytology, total protein and glucose were monitored routinely in the CSF.

### Pharmacokinetic sampling and processing

Plasma and CSF concentrations of temozolomide were analyzed in sparse samples collected on pre-defined timepoints, depending on the treatment schedule of the children. The exact time of temozolomide administration as well as the time of blood and CSF sampling were carefully documented.

Venous blood samples (5 ml) were drawn into cooled lithium-heparinized tubes at different time points during treatment with temozolomide. After collection, samples were immediately centrifuged (2000 rpm during 10 min at 4 °C), the plasma was then acidified with 0.1 ml 1 M HCl and stored immediately at  – 20 °C until analysis. CSF was collected via the Ommaya reservoir at different time points. The collected CSF was used for routine analysis, the last portion was taken for pharmacokinetic studies. Processing of the CSF samples was analogous to the blood samples but did not require centrifugation.

### Analysis of blood and CSF samples

The concentration of temozolomide in human plasma and CSF was determined by high-performance liquid chromatography (HPLC) using a Dionex UltiMate 3000 system (Thermo Fisher Scientific, Inc., Waltham, MA) with UV detection at 330 nm. Frozen plasma and CSF samples were thawed at room temperature. After the addition of 10 µl ice-cold trifluoroacetic acid to 190 µl plasma and CSF, the samples were centrifuged (13,000 g for 5 min at 4 °C) and 100 µl of the supernatant were injected onto a Hypersil BDS C18 column (5 µm, 250 × 4.6 mm I.D., Thermo Fisher Scientific, Waltham, MA) preceded by a Hypersil BDS-C18 guard column (5 µm, 10 × 4.6 mm I.D.) at a flow rate of 1 ml/min. The column oven was at 45 °C. The mobile phase consisted of a continuous gradient mixed from 0.1 formic acid in (mobile phase A) and acetonitrile (mobile phase B). Mobile phase B linearly increased from 0% (0 min) to 7.5% at 9 min, further increased to 80% at 9.5 min and was kept constant at 80% until 13 min. The percentage of acetonitrile was then decreased to 0% within 1 min to equilibrate the column for 6 min before injecting the next sample. Linear calibration curves were generated by spiking drug-free human plasma and CSF with standard solution of temozolomide to obtain a concentration range of 0.005 to 10 µg/ml (average correlation coefficients > 0.999). For this method, the limit of quantification in plasma and CSF was determined to be 5 ng/ml (coefficients of accuracy and precision were < 9%).

### Model-based pharmacokinetic analysis

The population pharmacokinetic model of temozolomide was developed using the nonlinear mixed-effects modeling program NONMEM (Version 7.4.2), PsN 4.8.1, and Pirana 2.9.9.

R (Version 4.0.3) was used to build figures for model evaluations and for statistical summaries. Estimations were performed using the first-order conditional estimation algorithm (FOCE) with interaction.

Different structural models with two and three compartments with or without transit compartments were tested during the model building process to fit temozolomide concentrations in plasma and CSF.

Model selection criteria were the objective function value (OFV) for nested models (a difference of ≥ 3.84 points was considered as statistically significant, corresponding to *p* < 0.05 with one degree of freedom), the Akaike information criterion (AIC) and Bayesian information criterion (BIC) for non-nested models, improvement in goodness-of-fit (GOF) plots, and the precision of final parameter estimates, assessed by nonparametric bootstrap analysis with 1000 samples [22].

Visual predictive checks (VPCs) were performed to evaluate different models by comparing observations from the original dataset with predicted concentrations from 1000 simulated datasets [23].

Inter-individual variability was tested on all pharmacokinetic parameters (assuming a log-normal distribution) and was included in the model if the OFV dropped by at least 3.84 points.

Inter-occasion variability was not implemented in the model. Proportional, additive, and combined error models of residual unexplained variability were tested.

The volume of distribution in the CSF (V_CSF_) was fixed to 90 ml during the modeling process, which reflects the mean of age-expected volumes (3 mL/kg in children and 2 mL/kg in adolescents) in our patient population [24].

A sensitivity analysis was performed to investigate the influence of different CSF volumes (range applicable for children and adolescents: 50–220 ml) on final parameter estimates and CSF penetration of temozolomide [25].

A bioavailability of 1 was assumed for temozolomide, corresponding to its known high bioavailability of nearly 100% after oral administration [3].

Age, weight, height, and body surface area (BSA) were considered as covariates on pharmacokinetic parameters of temozolomide.

BSA was calculated using the Mosteller formula [26] and all covariates were centered by the respective median values.

First, each covariate was added individually to the base model. A covariate was considered as significant if the OFV decreased by at least 3.84 points and if it produced a reduction in the parameters inter-individual variability. Subsequently, all significant covariates were ranked. In case covariates were correlated to each other (correlation coefficient > 0.5), only one covariate could be included in the model. Selection criteria were significance, biological plausibility, and improvement of GOF plots / VPCs.

### Model-based simulations and CSF penetration of temozolomide

Simulations were performed based on the final population pharmacokinetic model of temozolomide using NONMEM (Version 7.4.2), PsN 4.8.1, Pirana 2.9.9, and R 4.0.3.

A new dataset containing 11 virtual subjects with same demographics as the patients in the original dataset was created. For the simulations, each virtual subject received the same daily dose of temozolomide as the corresponding patient in the original dataset for a period of 7 days. Plasma and CSF sampling times were simulated every 12 min over the entire period to reach steady state.

Based on the parameter estimates obtained from the final population pharmacokinetic model of temozolomide, stochastic simulation runs were repeated 500 times for each virtual subject.

The AUC in plasma, the AUC in CSF, and the AUC_CSF_ / AUC_plasma_ ratio were calculated for each run at steady state from 144 to 168 h. The median AUC_CSF_ / AUC_plasma_ ratio was used to quantify CSF penetration of temozolomide.

## Results

### Demographics and treatment

Pharmacokinetic studies were performed in a total of 11 patients. Eight patients were male, three patients were female. Median age was 9 years (range 4–14 years).

Six patients suffered from recurrent medulloblastoma, and five patients had recurrent ependymoma. Demographic characteristics are shown in Table 1.

**Table 1:** Summary of demographic and clinical characteristics of the patients

Characteristic	Number of patients
Gender (male/female)	8/3
*Diagnosis*	
Recurrent medulloblastoma	6
Recurrent ependymoma	5
	Median (range)
Age (years)	9.5 (4–14)
Weight (kg)	26.65 (19.4–58)
Height (cm)	128 (101.3–163)
BSA^a^ (m^2^)	0.98 (0.77–1.62)

^a^*BSA* body surface area = (height (cm) × weight (kg) / 3600)^0.5^

All patients received a metronomic schedule with daily doses of temozolomide ranging from 24 to 77 mg/m^2^. All patients were treated with the metronomic regimen until disease progression.

### Temozolomide concentrations in plasma and CSF

In total, 28 plasma samples and 64 CSF samples were collected for the population pharmacokinetic analysis. Median temozolomide concentrations (range) were 0.96 (0.24–5.99) µg/ml in plasma, and 0.37 (0.06–1.76) µg/ml in CSF.

The median (range) number of samples per patient was 2 (0–8) in plasma and 4 (1–14) in CSF.

**Fig. 1:**
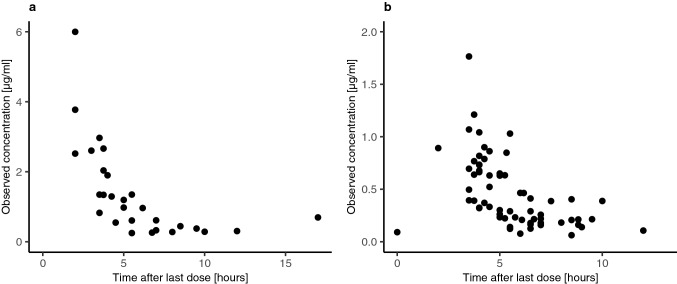
Observed concentration–time profiles of temozolomide in plasma (**a**) and CSF (**b**)

Figure 1 shows the observed concentration–time profiles of temozolomide in plasma and CSF.

### Population pharmacokinetic analysis

**Fig. 2:**
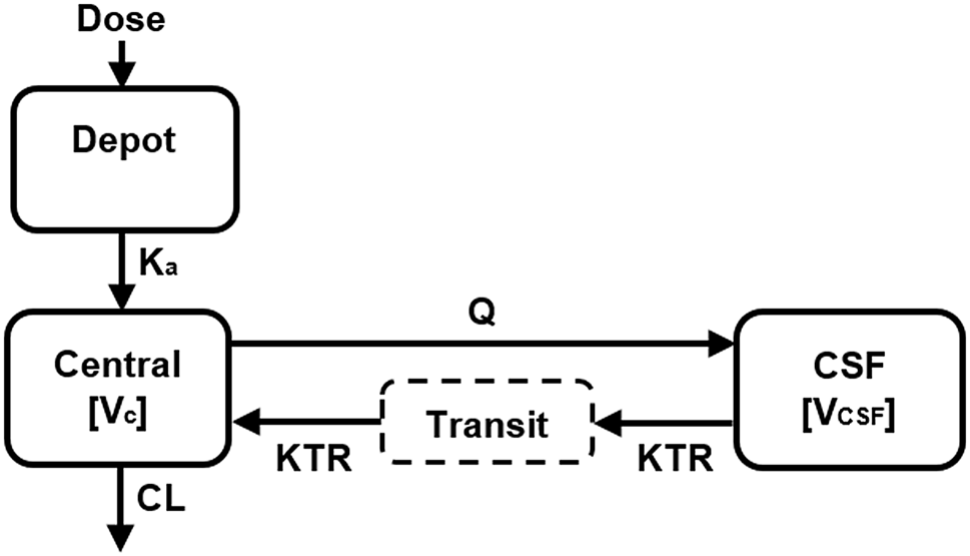
Structure of the final two-compartment model for temozolomide. *K*_*a*_ first-order absorption rate constant, *CL* clearance, *V*_*c*_ volume of distribution in the central compartment, *CSF* cerebrospinal fluid, *V*_CSF_ volume of distribution in the CSF, *Q* intercompartmental clearance between the central compartment and the CSF, *KTR* transit rate

A two-compartment model (central/plasma [1], CSF [2]) with first-order absorption, first-order elimination, and a transit compartment between CSF and plasma adequately described temozolomide concentrations in plasma and CSF (Fig. 2). The implementation of an additional transit compartment between CSF and plasma decreased the OFV by 60.3 points, compared to a two-compartment model without a transit compartment (decrease in the OFV from -128.3 to -188.6 points). The addition of a third compartment did not result in a significant improvement in the OFV, and worsened model stability.

An exponential model was used to describe the inter-individual variability in clearance (CL), however, the estimation of inter-individual variability on the absorption rate constant (K_a_), the volume of distribution in the central compartment (V_c_), the intercompartmental clearance (Q), and the transit rate between CSF and the central compartment (KTR) did not improve the model significantly. The residual unexplained variability was best described by a combined error model.

Weight (dOFV -15.8), age (dOFV -21.7), height (dOFV -27.2) and BSA (dOFV -27.5) were statistically significant covariates on CL. All significant covariates were highly correlated to each other with correlation coefficients between 0.83 and 0.99. BSA was retained in the model because it reflects weight and height and performed best in terms of improvement of the OFV, GOF plots and VPCs.

The final covariate model on CL was therefore represented by TVCL = θ_1_ * (BSA/0.98), where TVCL is the typical value of CL.

Ignoring the correlation between the significant covariates, a stepwise covariate modelling approach using forward inclusion (*p* < 0.05) and backward elimination (*p* < 0.001) of significant covariates led to the same final covariate model.

Table 2 shows the population estimates and bootstrap medians with respective 95% confidence intervals (CI) of the final population pharmacokinetic model of temozolomide in plasma and CSF.

**Table 2:** Parameter estimates of temozolomide obtained from the final model

Parameter	Point estimate	Bootstrap median (95% CI^a^)	RSE%^i^
*Fixed effects*			
K_a_^b^ (h^−1^)	9.64	9.55 (6.94–11.90)	13.33
CL^c^ (liter/h)	3.29	3.14 (2.58–3.95)	10.46
V_c_^d^ (liter)	10.5	10.54 (8.17–14.32)	15.82
V_CSF_^e^ (liter)	0.09 FIXED		
Q^f^ (liter/h)	0.0327	0.031 (0.014–0.038)	18.21
KTR^g^ (h^−1^)	0.983	0.98 (0.534–1.22)	15.23
*Inter-individual variability* *(CV%*^h^*)*			
CL	8.3%	8.2 (0.84–26.0)	150.02
*Residual unexplained variability*			
Proportional error, plasma	0.0911	0.0837 (0.005–0.169)	43.54
Additive error, plasma	0.0722	0.0717 (0.004–0.157)	55.48
Proportional error, CSF	0.126	0.125 (0.035–0.204)	35.78
Additive error, CSF	0.0019	0.0019 (0.000003–0.0055)	96.23

^a^*CI* confidence interval

^b^*K*_*a*_ absorption rate constant for first-order absorption

^c^*CL* clearance

^d^*V*_*c*_ volume of distribution in the central compartment

^e^*V*_CSF_ volume of distribution in the CSF

^f^*Q* intercompartmental clearance

^g^*KTR* transit rate between CSF and the central compartment

^h^*CV*% coefficient of variation in percent

^i^*RSE*% relative standard error in percent

### Model evaluation

**Fig. 3:**
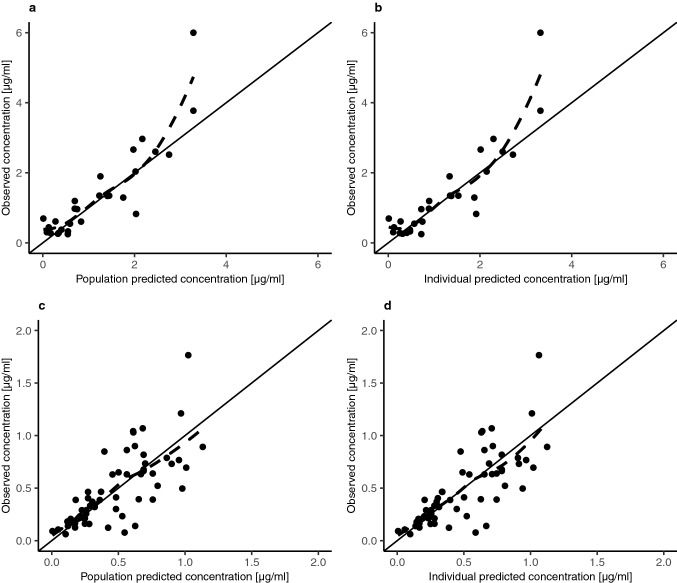
Goodness-of-fit plots for the final model. Population predicted concentrations versus observed concentrations in plasma (**a**) and CSF (**c**). Individual predicted concentrations versus observed concentrations in plasma (**b**) and CSF (**d**). Solid black lines represent lines of identity, dashed lines indicate locally weighted smoothing lines

**Fig. 4:**
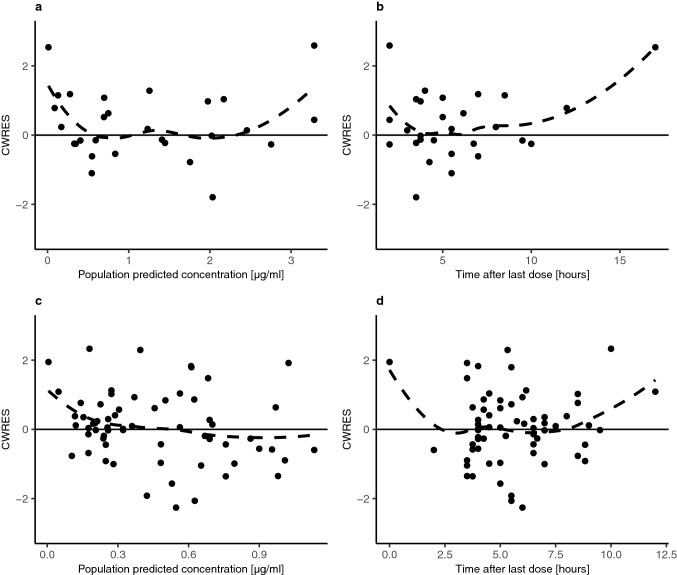
Goodness-of-fit plots for the final model. Population predicted concentrations versus conditional weighted residuals in plasma (**a**) and CSF (**c**). Time after last dose versus conditional weighted residuals in plasma (**b**) and CSF (**d**). Solid black lines represent lines of zero residuals, dashed lines indicate locally weighted smoothing lines

**Fig. 5:**
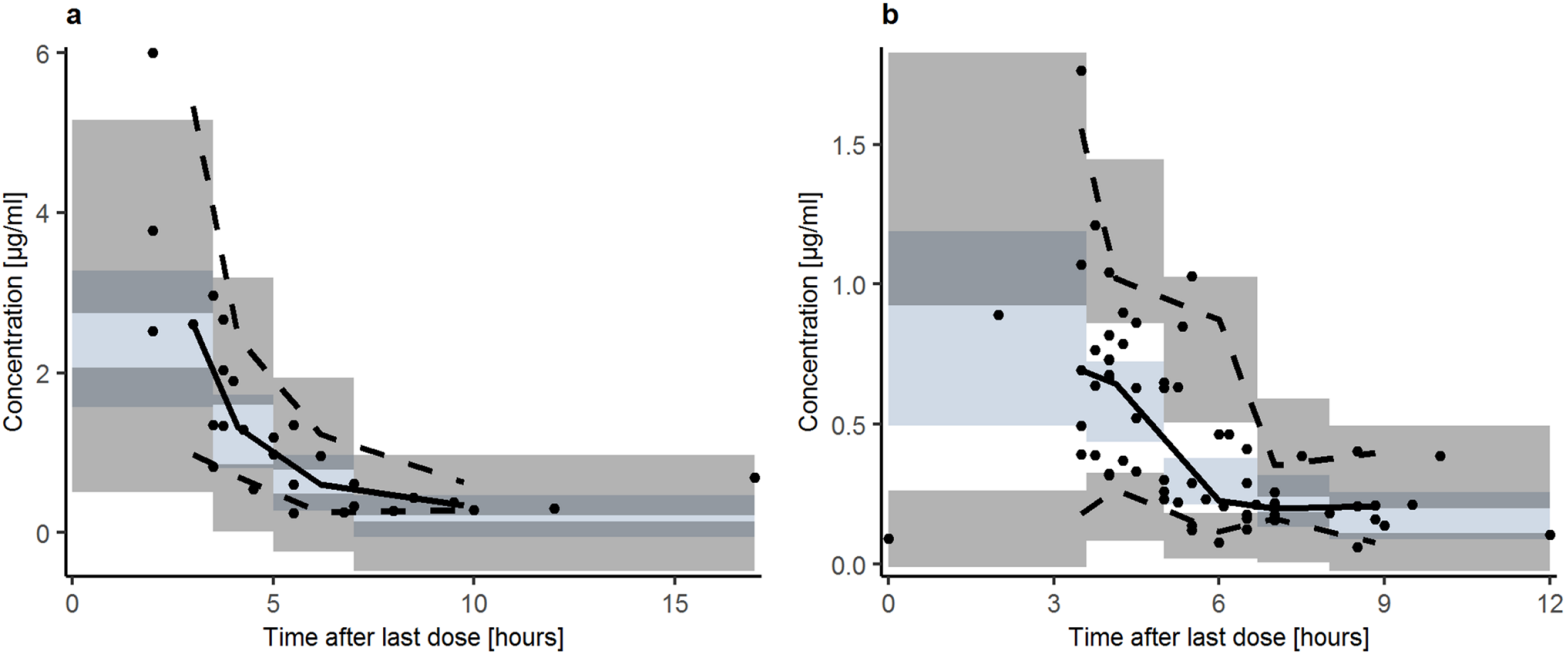
Confidence interval visual predictive check (*n* = 1000) for the final model in plasma (**a**) and CSF (**b**). Solid dots represent observed concentrations. Black solid lines represent the median while dashed lines show the 5th and 95th percentile of observed concentrations. Shaded areas are the model-predicted 95% CIs for the median (blue), and 5th and 95th percentile (grey) from 1000 simulated datasets

GOF plots showed that the model described the data reasonably (Figs. 3 and 4). There was good agreement between observed and individual / population predicted concentrations in plasma and CSF. Since the data supported inter-individual variability only on CL, differences between individual and population predicted concentrations were small. Conditional weighted residuals (CWRES) were randomly scattered around zero, indicating no systematic deviations in the model. The comparison between individual concentration–time profiles and model predictions for all 11 patients in plasma and CSF demonstrated good fit (Online Resource 1). VPCs showed that the model captured the central tendency and spread of the observed data in plasma and CSF, however, a slight underprediction for late concentrations in plasma was visible (Fig. 5). The bootstrap results indicated good stability of the final parameter estimates.

All parameter estimates were close to the bootstrap median values, and all estimates were within the respective 95% CI (Table 2).

All in all, GOF plots, VPCs and bootstrap analysis demonstrated that the final model was able to describe the data appropriately.

### Temozolomide penetration into the CSF

The median AUC (95% prediction interval) at steady state for all 11 patients was 16.21 (6.82–22.22) mg*h/L in plasma and 5.99 (2.52–8.21) mg*h/L in CSF. The median AUC_CSF_/AUC_plasma_ ratio was 37%. As mentioned above, the data did not support the estimation of inter-individual variability on other pharmacokinetic parameters than CL. In the absence of variability on parameters that describe the transfer of temozolomide between plasma and CSF (Q and KTR), a meaningful prediction interval for the AUC_CSF_/AUC_plasma_ ratio cannot be provided, because the ratio is constant among all individuals at steady state.

The sensitivity analysis showed that the use of different CSF volumes had no significant influence on the final parameter estimates. The median AUC_CSF_/AUC_plasma_ ratio of temozolomide decreased to 31% and 32% for CSF volumes of 50 and 220 ml, respectively.

## Discussion

Temozolomide is frequently administered as a standard agent for pediatric patients with CNS tumors [27, 28], but little is known so far about the pharmacokinetics and the exposure of temozolomide in CSF, as compared to plasma concentrations in the pediatric population.

Especially in recurrent CNS tumors in children, a metronomic schedule is frequently used as a therapeutic option, which means the continuous application of chemotherapy in low doses without treatment-free intervals [29, 30].

The present analysis was a comprehensive evaluation of metronomic oral temozolomide dosing in pediatric patients with malignant CNS tumors by population pharmacokinetic modeling and simulations.

A structural two-compartment model (central/plasma [1], CSF [2]), with first-order absorption and first-order elimination, best described the pharmacokinetics of temozolomide in plasma and CSF in our 11 pediatric CNS tumor patients, which were treated according to a low dose metronomic schedule with daily doses between 24 and 77 mg/m^2^. This is in accordance with a previously published model in the adult population [5]. However, in our model, an additional transit compartment was required to describe the transfer of temozolomide between CSF and plasma. Several studies assessed the plasma pharmacokinetics of temozolomide in the pediatric population across different dose levels. Observed mean values in two noncompartmental analyses ranged from 4.32 to 8.19 l/h/m^2^ for temozolomide CL, and from 7.78 to 21.99 l/m^2^ for the volume of distribution [7, 9]. In a population pharmacokinetic analysis of temozolomide in 39 children with primary CNS tumors, population estimates (range) for temozolomide CL and the volume of distribution for a one-compartment model were 4.9 (1.6–10.8) l/h/m^2^ and 12.6 (1.5–29.3) l/m^2^, respectively [10]. After normalization to BSA, the population estimate obtained for V_c_ in our analysis (10.4 l/m^2^) is consistent with results from the previous studies. However, our normalized population estimate (range) of 3.34 (3.00–3.61) l/h/m^2^ for temozolomide CL is lower than previously reported values in the pediatric population. Panetta et al. observed a significant relationship between the administered dose of temozolomide and apparent temozolomide CL. On average, a 17% decrease in temozolomide CL was observed when the administered dose was reduced from 200 to 150 mg/m^2^ per day [10].

In comparison, temozolomide doses administered in our population range from 24 to 77 mg/m^2^ per day. In addition to the high variability observed in individual temozolomide CL values by Panetta et al., this may also serve as an explanation for the differences in apparent temozolomide CL values between our study and previous reports.

Our population estimate for K_a_ (9.64 h^−1^) is larger than values previously found in adults (5.8 h^−1^ CV%: 111%) [5], and children (2.4 h^−1^ range: 0.14–31.6) [10]. This deviation might be explained by the lack of earlier blood samples after administration of temozolomide, which makes it difficult to estimate K_a_ in our population, and by the observed substantial variability concerning this parameter in the previous studies. As reported in other population pharmacokinetic studies of temozolomide in adults and children, we could identify age, weight, height, and BSA as significant covariates on temozolomide CL [5, 10].

To date, it has not been assessed directly to which extent oral metronomic temozolomide can reach the CSF and/or penetrate the brain tissue, and whether therapeutically effective concentrations of temozolomide in CSF can be achieved in pediatric patients. To further evaluate this important clinical issue, we developed and applied a new model system to quantitatively measure temozolomide in serum and CSF of pediatric patients who received oral metronomic temozolomide for treating their disease. Overall, we found a median plasma level of temozolomide of 0.96 µg/ml, compared with a median CSF level of 0.37 µg/ml in our pediatric CNS tumor patients.

The AUC values in our analysis revealed CSF exposure corresponding to 37% of that observed in plasma. Our results show higher levels in children than the values reported in previous adult studies, where CSF levels of temozolomide of about 20% have been found, when compared to the plasma levels [5].

Some evidence exists that permeability of the BBB is increased in patients who were treated with cranial radiotherapy [31]. Ostermann et al. observed a trend towards a 15% increase in the transfer of temozolomide from plasma to CSF in case of concomitant radiotherapy in the adult population [5]. All children in our study had received previous treatment with cranial radiotherapy. This might therefore be one reason for the higher penetration of temozolomide into the CSF in our pediatric study cohort. As compared to previous evaluations, all patients in our study also received concomitant intraventricular chemotherapy which may also have influenced the permeability of the blood-CSF barrier and thereby the penetration of temozolomide into the CSF. Our results show similar AUC_CSF_ / AUC_plasma_ ratios as obtained from nonhuman primate models for temozolomide (33% ± 6%) [4].

The present study has some limitations. First, our patient population was relatively small (*n* = 11), and sampling was sparse (28 plasma samples, 64 CSF samples). This limited our ability to estimate inter-individual variability on pharmacokinetic parameters other than CL. We were not able to determine variability in CSF penetration of temozolomide at steady state, and we could not investigate factors that might influence CSF penetration. The study was planned as a real-world investigation, so the collection of plasma and CSF samples could not strictly follow a predetermined schedule. The samples were essentially taken during routine examinations to avoid additional discomfort and risk for study patients.

Second, it must be considered that temozolomide is a prodrug and not the biologically active methylating agent itself. Temozolomide is spontaneously hydrolyzed to 5-(3-methyltriazen-1-yl) imidazole-4-carboxamide (MTIC) at physiological pH, which then exerts the antitumor activity [32]. The concentration of the active agent MTIC in the CSF has not been quantified in this study, which is not feasible in a clinical study due to the short half-life of only 2 min [33]. The AUC_CSF_/AUC_plasma_ ratio was used to measure CSF penetration of temozolomide in the current analysis. The ratio serves as a surrogate parameter to describe how effectively a chemotherapeutic agent (e.g., temozolomide) can be transported into the CNS. However, CSF penetration cannot be equated to brain and/or tumor tissue penetration, and any conclusions to this end must be made with caution because of differences in the blood-CSF-, blood–brain-, and blood–brain-tumor-barrier, even though temozolomide has shown comparable results regarding the penetration into the CSF and brain tissue in previous studies in the adult population [5, 11, 34].

Third, V_CSF_ was fixed to its expected mean value (90 ml) in our pediatric population because any attempt to estimate V_CSF_ did not improve the model, and worsened model stability. However, a sensitivity analysis showed an essentially similar CSF penetration for CSF volumes of 50 and 220 ml, compared to a volume of 90 ml.

Fourth, our final population pharmacokinetic model of temozolomide in plasma and CSF was only internally validated, and no external validation was performed because of the limited number of patients.

Taken together, we present for the first time evidence that metronomic oral temozolomide penetrates into the CSF in pediatric patients, with even higher concentration levels compared to adults.

Our results provide a pharmacokinetic rationale to support the concept of oral metronomic temozolomide in malignant pediatric CNS tumors.

Moreover, validation of our results may also lead to a better understanding of temozolomide pharmacokinetics in CSF and serum, and enable better treatment strategies for brain tumor patients, especially in the recurrent setting.

## Supplementary Information

Below is the link to the electronic supplementary material.

Supplementary file1 (PDF 299 KB)

## Data Availability

All data generated or analysed during this study are included in this published article (and its supplementary information files).
