# Supplementary material for: Pharmacokinetics of metronomic temozolomide in cerebrospinal fluid of children with malignant central nervous system tumors
Source: Cancer Chemother Pharmacol. 2022 Mar 30;89(5):617–27. doi: 10.1007/s00280-022-04424-4 (PMC9054874; doi:10.1007/s00280-022-04424-4)
Supplement: Supplementary file 1 — Supplementary file1 (PDF 299 KB) [file 280_2022_4424_MOESM1_ESM.pdf]

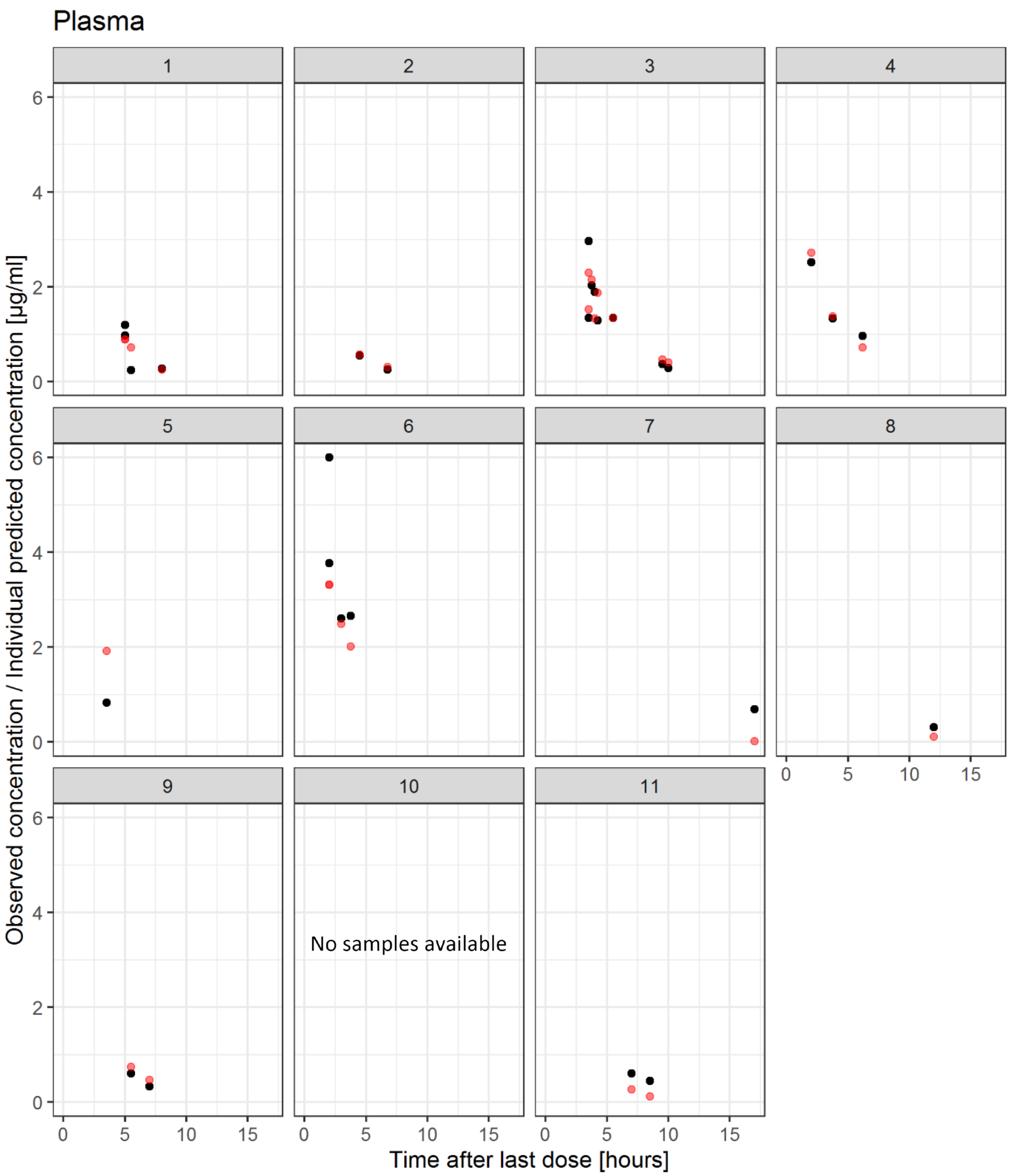

Supplementary Fig. 1 Observed concentrations (black dots) and individual predicted concentrations (red dots) versus time after last dose for each patient in plasma.

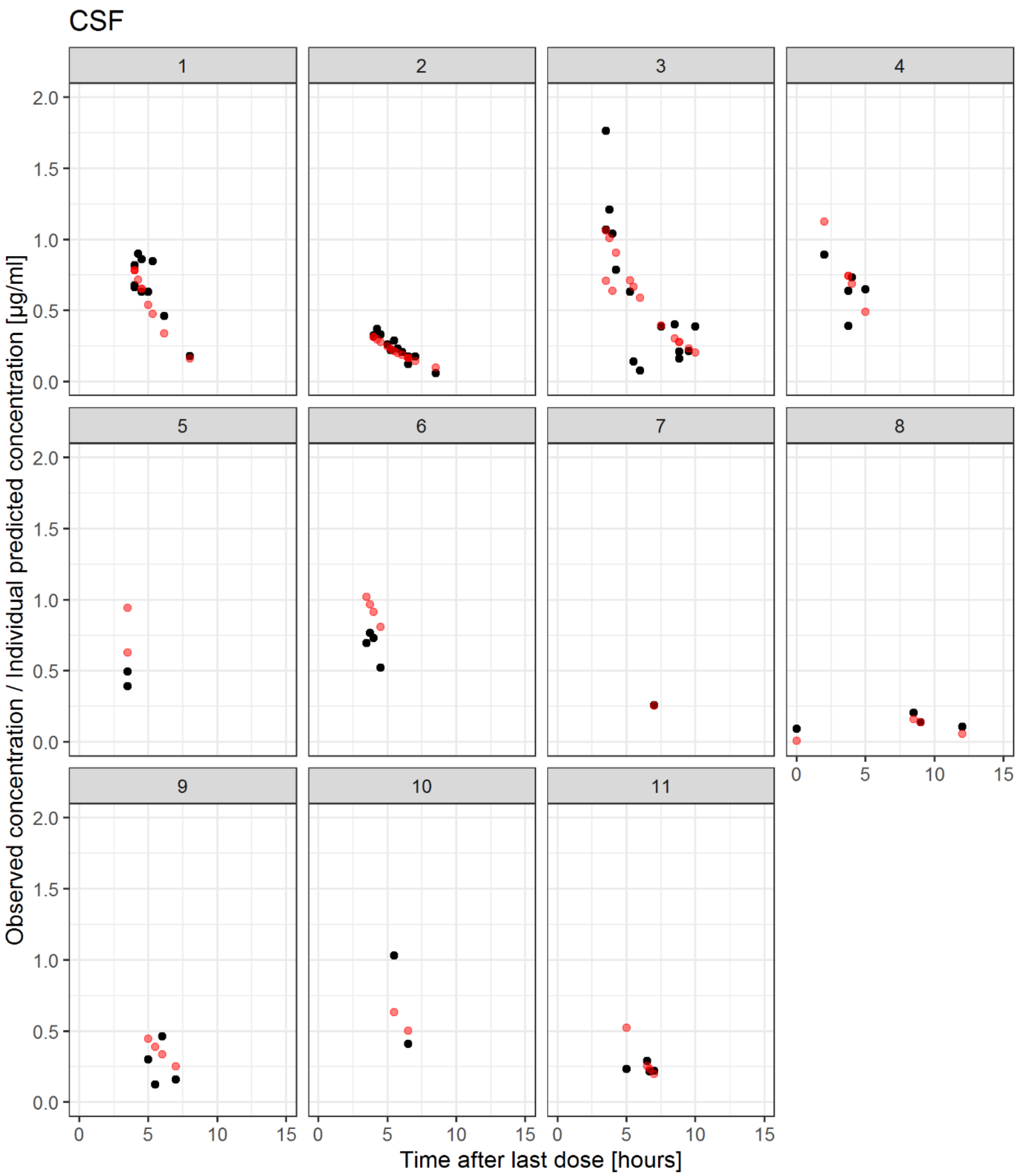

Supplementary Fig. 2 Observed concentrations (black dots) and individual predicted concentrations (red dots) versus time after last dose for each patient in CSF.
